# Coordinated Defects in Hepatic Long Chain Fatty Acid Metabolism and Triglyceride Accumulation Contribute to Insulin Resistance in Non-Human Primates

**DOI:** 10.1371/journal.pone.0027617

**Published:** 2011-11-18

**Authors:** Subhash Kamath, Alberto O. Chavez, Amalia Gastaldelli, Francesca Casiraghi, Glenn A. Halff, Gregory A. Abrahamian, Alberto M. Davalli, Raul A. Bastarrachea, Anthony G. Comuzzie, Rodolfo Guardado-Mendoza, Lilia M. Jimenez-Ceja, Vicki Mattern, Ana Maria Paez, Andrea Ricotti, Mary E. Tejero, Paul B. Higgins, Iram Pablo Rodriguez-Sanchez, Devjit Tripathy, Ralph A. DeFronzo, Edward J. Dick, Gary W. Cline, Franco Folli

**Affiliations:** 1 Department of Medicine/Division of Diabetes. The University of Texas Health Science Center at San Antonio, San Antonio, Texas, United States of America; 2 Institute of Clinical Physiology, National Research Council, Pisa, Italy; 3 The UT Transplant Center, The University of Texas Health Science Center at San Antonio, San Antonio, Texas, United States of America; 4 Department of Internal Medicine, Diabetes & Endocrinology Unit, San Raffaele Scientific Institute, Milano, Italy; 5 Southwest National Primate Research Center/Texas Biomedical Research Institute, San Antonio, Texas, United States of America; 6 Department of Internal Medicine, Yale University School of Medicine, New Haven, Connecticut, United States of America; University of Tor Vergata, Italy

## Abstract

Non-Alcoholic fatty liver disease (NAFLD) is characterized by accumulation of triglycerides (TG) in hepatocytes, which may also trigger cirrhosis. The mechanisms of NAFLD are not fully understood, but insulin resistance has been proposed as a key determinant.

**Aims:**

To determine the TG content and long chain fatty acyl CoA composition profile in liver from obese non-diabetic insulin resistant (IR) and lean insulin sensitive (IS) baboons in relation with hepatic and peripheral insulin sensitivity.

**Methods:**

Twenty baboons with varying grades of adiposity were studied. Hepatic (liver) and peripheral (mainly muscle) insulin sensitivity was measured with a euglycemic clamp and QUICKI. Liver biopsies were performed at baseline for TG content and LCFA profile by mass spectrometry, and histological analysis. Findings were correlated with clinical and biochemical markers of adiposity and insulin resistance.

**Results:**

Obese IR baboons had elevated liver TG content compared to IS. Furthermore, the concentration of unsaturated (LC-UFA) was greater than saturated (LC-SFA) fatty acyl CoA in the liver. Interestingly, LC-FA UFA and SFA correlated with waist, BMI, insulin, NEFA, TG, QUICKI, but not M/I. Histological findings of NAFLD ranging from focal to diffuse hepatic steatosis were found in obese IR baboons.

**Conclusion:**

Liver TG content is closely related with both hepatic and peripheral IR, whereas liver LC-UFA and LC-SFA are closely related only with hepatic IR in non-human primates. Mechanisms leading to the accumulation of TG, LC-UFA and an altered UFA: LC-SFA ratio may play an important role in the pathophysiology of fatty liver disease in humans.

## Introduction

Non-Alcoholic Fatty Liver Disease (NAFLD) is the most common hepatic disease in both adults and children worldwide. NAFLD may progress from steatosis to steatohepatitis (NASH), advanced fibrosis and cirrhosis [1]. It is strongly associated to components of the metabolic syndrome including obesity, insulin resistance, dyslipidemia and type 2 diabetes mellitus (T2DM) [2], and it is estimated that up to one third of the general population in western societies may have hepatic steatosis [3]. Interestingly, various degrees of liver steatosis are found in ∼75% of obese subjects and virtually in 100% of individuals with T2DM. NAFLD is also associated with an increase in the risk of cardiovascular disease and liver related all cause mortality [4], [5], [6].

Previous studies have shown that the main predictors of NAFLD are increased BMI, waist circumference, triglyceride, and GGT or ALT concentrations, but also that NAFLD is associated with an increased AST/ALT ratio, insulin resistance, the metabolic syndrome and T2DM [7], [8]. It has been proposed that insulin resistance in skeletal muscle, liver and adipose tissue may play a concerted role in the pathogenesis of NAFLD [7], [9].

Hepatic triglyceride accumulation is proportional to hepatic insulin resistance and subjects with NAFLD/NASH have increased circulating NEFA due to increased lipolysis and impaired suppression of NEFA release both during clamp and after a glucose load [10], [11]. Alarmingly, NAFLD is present in obese children that often have not only steatosis but also have fibrosis with NASH [11], [12], [13].

The current knowledge about the cellular and molecular defects of liver steatosis and fatty acid metabolism has mainly relied upon the study of rodent models [14], [15], [16]. We have recently described the baboon (*Papio hamadryas sp.*) as a natural non-human primate model for the study of insulin resistance and T2DM. Non-human primates develop features characteristic of the metabolic syndrome, including obesity, insulin resistance and T2DM with insulin signaling and insulin secretory molecular defects similar to humans [17], [18], [19], [20]. Moreover, previous studies of gene expression profiles using cDNA arrays have shown a great deal of similarity to humans in the differential expression of several novel genes and pathways in diverse tissues, and these similarities are highly preserved at the protein level [21]. Here, we characterized the profile of fatty acid accumulation in liver from obese insulin resistant baboons by direct measurements of liver TG content and their association with clinical indexes of central (hepatic) and peripheral (muscle) insulin sensitivity/resistance.

## Methods

### Ethics Statement

This study was performed in strict accordance with the recommendations in the Guide for the Care and Use of Laboratory Animals of the National Institutes of Health. The protocol was approved by the Institutional Animal Care and Use Committee of the Texas Biomedical Research Institute (Assurance Number: A3082-01). The study approval ID is IACUC 923 PC. After an overnight fast (∼12 h), each baboon was sedated with ketamine hydrochloride (10 mg/kg i.m.) before arrival in the procedure room. Endotracheal intubation was performed using disposable cuffed tubes (6.5–8.0 mm diameter) under direct laryngoscopic visualization, and all the animals were supported with 98–99.5% FiO2 by a pressure controlled ventilator adjusted, as necessary, to keep the oxygen saturation >95%. The maintenance of anesthesia consisted of an inhaled isofluorane (0.5–1.5%) and oxygen mix and every effort was made to minimize suffering.

### Animals

Twenty nondiabetic baboons, 10 males and 10 females, ranging over a wide span of BMI (16.5–39.5 kg/m^2^) were studied. Of note, in our facility baboons are gang housed, fed *ad libitum* with a standard monkey chow diet ([22] (Table S1), and have unrestrained physical activity. We performed clinical and biochemical characterization, assessment of body composition by DXA and insulin sensitivity using a euglycemic hyperinsulinemic clamp (60 mU/m^2^•min^−1^) including liver biopsies prior to the clamp in the whole group. The study population was highly representative of the whole baboon colony, and the clinical and biochemical characterization of the model and screening algorithms have been published elsewhere [17], [18]. The animals were not euthanized at the end of the euglycaemic clamp with biopsies. They were recovered, given appropriate veterinary care in the animal hospital for two days after the clamp and were released in the baboon colony.

### Evaluation of Skeletal Muscle and Liver Insulin Sensitivity

In order to evaluate hepatic insulin sensitivity we used the Quantitative Insulin-sensitivity Check Index (QUICKI) that primarily reflects liver glucose metabolism under fasting conditions. Also, we quantitated the insulin stimulated rate of glucose uptake (mainly reflecting skeletal muscle) with the hyperinsulinemic euglycemic clamp as previously described [17].

During the euglycemic insulin clamp, peripheral insulin sensitivity which primarily reflects muscle glucose uptake, was calculated as the mean rate of insulin-stimulated whole body glucose disposal (M) during the 90–120 min time period, since at this high level of hyperinsulinemia endogenous glucose production is expected to be completely suppressed even in insulin resistant animals [23]. To account for differences in circulating insulin levels among the subjects, peripheral insulin sensitivity was expressed as M/I, where I is the steady state plasma insulin at the end of the clamp.

Since fasting glucose concentrations are mainly determined by hepatic glucose output after an overnight fasting period, the use of indices employing fasting plasma insulin and fasting plasma glucose concentrations (i.e. HOMA-IR and QUICKI) provide an estimate of insulin sensitivity in the liver. They provide an alternative measure to the direct measurement of hepatic insulin sensitivity by determination of glucose production with glucose turnover studies and tracer methodology, when this gold standard technique is not available. Thus, we estimated hepatic insulin sensitivity by QUICKI as 1/[log (Fasting Plasma Insulin)+log (Fasting Plasma Glucose)], (FPI, FPG) [24], [25]. Hepatic insulin clearance was measured as previously reported [10]. Additionally, in order to further characterize insulin sensitivity profiles in peripheral tissues in our study population we calculated the adipose tissue insulin resistance index as the product of fasting NEFA×FPI, since insulin suppresses lipolysis and NEFA delivery to circulation by adipocytes [23].

### Analytical Determinations

Plasma glucose was measured by the glucose oxidase method using a Beckman Glucose Analyzer 2 (Beckman-Coulter, Fullerton, CA). Chemistry panels including liver enzymes were measured with commercial kits in an ACE Clinical Chemistry System (Alfa Wassermann Diagnostic Technologies, NJ), and plasma insulin was measured by radioimmunoassay (Diagnostic Products, Los Angeles, CA). Leptin was measured at baseline by radioimmunoassay from frozen plasma aliquots as per manufacturer's protocol (Millipore, St. Charles, MO). IGF-1 levels were measured with Immulite, as per manufacturer's protocol (Siemens Healthcare Diagnostics, Deerfield, IL, USA).

### Liver Tissue, Histology and NAFLD Scoring System

Liver biopsies were collected at 0′–30′–120′ during the clamp, snap frozen in liquid nitrogen and stored at −80°C until further use. Liver sections were placed in 10% neutral phosphate buffered formalin, fixed overnight, processed conventionally, embedded in paraffin, cut at 5 µm and stained with hematoxylin and eosin (H&E) and Masson's trichrome using standard laboratory methods. A board certified veterinary pathologist (E.J.D) who was blinded to clinical information and sequence of biopsy scored each biopsy for steatosis, lobular inflammation, hepatocellular ballooning, and fibrosis using the NAFLD Activity Score (NAS) and Fibrosis Staging System [26].

### Liver Triglyceride Measurement

Lipid extracts from previously frozen liver tissue were assayed for triglycerides according to the manufacturer's protocol using the triglyceride quantification kit (BioVision, Mountain View, CA). This assay is based on the principle that triglycerides are broken down to non-esterified fatty acids and glycerol by lipase. The glycerol is then oxidized to generate a reactive product that is detected by colorimetry (spectrophotometry at λ = 570 nm) and fluorescence (Ex/Em = 535/587 nm). Detection range of the assay is 2 pmol–10 nmol (or 2–10000 µM range) of triglyceride in various samples. Monoglycerides and diglycerides are also detected. Protein concentration was measured by the Bradford assay (Bio-Rad) to normalize triglyceride content in liver samples.

### Measurement of Hepatic Long Chain Fatty Acyl CoA

Liquid nitrogen frozen liver samples were homogenized in 100 mM KH_2_PO_4_, pH 4.9 and 2-propanol. Heptadecanoyl CoA was added as internal standard. Saturated (NH_4_)_2_SO_4_ and acetonitrile were added for phase separation solid phase extraction using Oligonucleotide Purification Cartridges (Applied Biosystems, Singapore). The cartridges were washed with distilled water, and long-chain fatty acyl CoA esters (LCFACoA) were eluted slowly with 0.5 ml of 60% acetonitrile. The eluent was dried, and then reconstituted in 100 µl of methanol/H_2_O for ESI/MS/MS analysis. Analysis was performed on a bench top tandem mass spectrometer API3000 (Perkin-Elmer Sciex) interfaced with a TurboIonspray ionization source in flow injection mode. Using negative electrospray ionization mode, LCFACoA are ionized predominantly to a doubly charged form and yields abundant specific product ions from CID (collision induced dissociation). LCFACoA are quantified by monitoring [M-2H]^2−^/[M-H-80]^−^as previously described [27]. Electrospray ionization of the LC-CoA esters was effected after separation and elution from a reverse-phase (C18) column (Table S2).

### Statistical analysis

Statistical calculations were performed with StatView for Windows, (version 5.0.1, SAS Institute, Inc., Cary, NC). All data are expressed as mean ± SE. Measured parameters found to have positive skewness were transformed to natural logarithms. Student *t*-test or Mann-Whitney U test were used for comparisons between insulin resistant and insulin sensitive baboons, where appropriate. Also, coefficients of correlations between metabolic/anthropometric variables, insulin sensitivity and liver triglycerides and LCFCoA were determined using the Pearson or Spearman's methods where applicable. Lastly, we used a step-wise method to perform a multivariate linear regression analysis to analyze the determinants of hepatic insulin sensitivity and liver triglyceride content.

## Results

**Figure 1:**
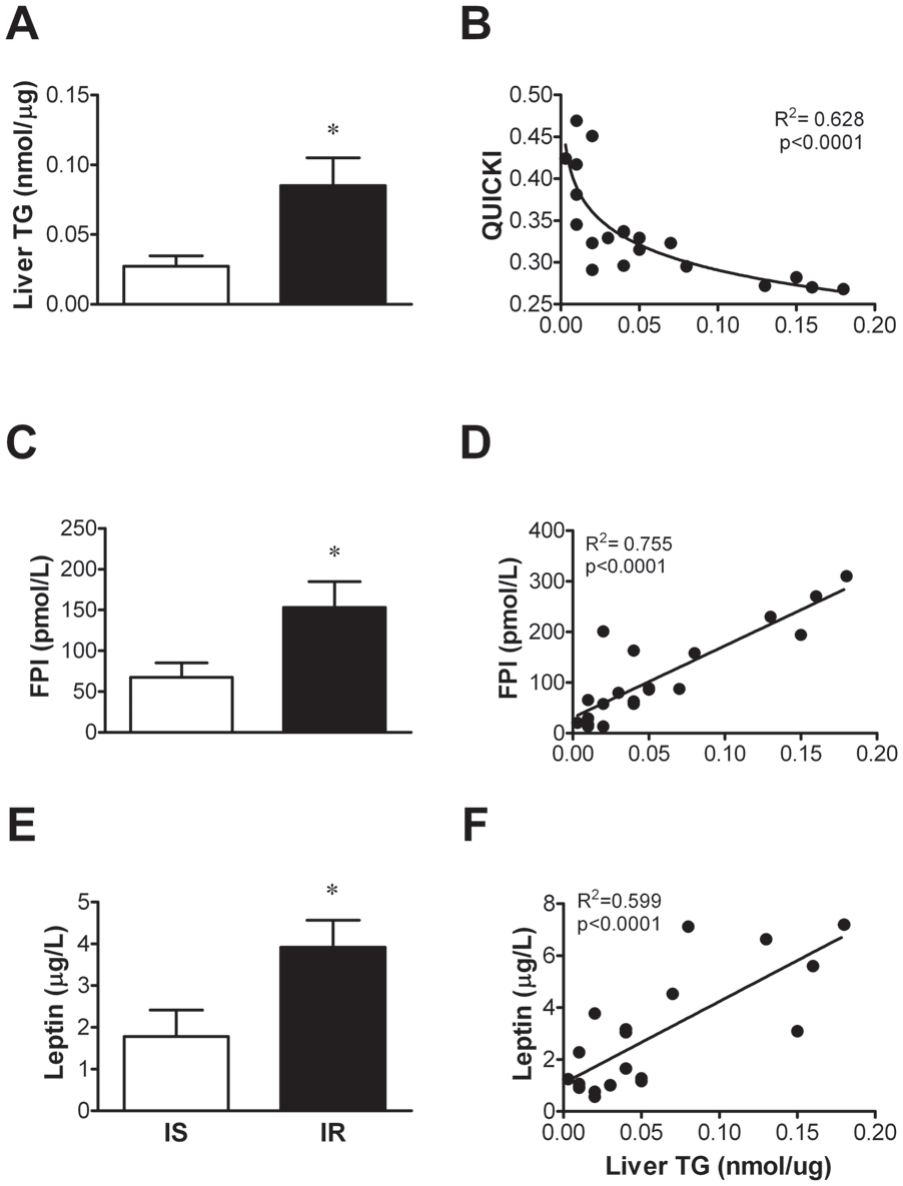
Liver TG content as a determinant of hepatic insulin resistance. Liver TG content in relation to insulin sensitivity level determined by the clamp (A) and correlation with estimates of hepatic insulin sensitivity – QUICKI (B), and fasting plasma insulin (C–D) and leptin (E–F) levels in baboons. *p<0.05.

Clinical and biochemical characteristics are summarized in Table 1. Insulin sensitivity and body fat composition were comprised into a wide range. Baboons were divided in two groups based on insulin sensitivity, those with insulin resistance (mean M = 2±0.3 mg/kg.min^−1^), and a control group of insulin sensitive animals (mean M = 11±0.7 mg/kg.min^−1^). Insulin resistant animals had increased adiposity (BMI, waist circumference and percent body fat) when compared to controls (all p<0.01). Fasting plasma insulin and leptin were significantly higher in the insulin resistant group (p<0.05, Figure 1). Moreover, there was a trend towards higher fasting plasma glucose, non-esterified fatty acids, hemoglobin A_1C_ and serum triglycerides concentrations in obese insulin resistant animals. No differences were found in ALT and AST concentrations between groups.

**Table 1:** Clinical and Biochemical characteristics of the study population.

Demographics/Morphometrics	Insulin resistant(n = 10)	Insulin sensitive(n = 10)	*P* value
Age (years)	21±0.8	18±0.7	NS (0.249)
Gender (M/F)	4/6	6/4	NS (0.398)
Waist (cm)	65±1	49±0.7	<0.0001
BMI	29±0.7	22±0.5	0.005
Body fat (%)	17±1	6±0.5	0.006
**Biochemistry**			
FPG (mmol/l)	6.1±0.05	5.5±0.1	NS (0.19)
HbA_1c_ (%)	4.9±0.3	4.5±0.2	NS (0.256)
FPI (pmol/l)	152.7±7	69.4±6	0.029
ALT (U/l)	31±1	28±1	NS (0.409)
AST (U/l)	29±1	30±1	NS (0.808)
Total cholesterol (mmol/l)	2.4±0.05	2.1±0.05	NS (0.466)
Triglycerides (mmol/l)	0.655±0.02	0.5±0.01	NS (0.137)
LDL-cholesterol (mmol/l)	0.85±0.02	0.93±0.05	NS (0.738)
HDL-cholesterol (mmol/l)	1.3±0.02	1±0.02	NS (0.075)
Leptin (µg/l)	3.9±0.4	1.7±0.4	0.029
F-NEFA (µmol/l)	635±150	441±170	NS (0.127)
**Indices of Insulin Resistance**			
AIRI	16.4±5	5.1±1.9	0.043
QUICKI	0.309±0.06	0.367±0.08	0.02
M (mg/kg•min^−1^)	2±0.3	11±0.7	<0.0001
M/I (mg/kg•min^−1^)/(mU/l)	1±0.2	5±0.5	<0.0001
Hepatic Insulin Clearance (ml/m^2^•min^−1^)	239±2	325±3	0.025

FPG = fasting plasma glucose; FPI = fasting plasma insulin; NEFA = non-esterified fatty acids; AIRI = adipocyte insulin resistance index; M/I = glucose uptake/steady state plasma insulin; QUICKI = quantitative insulin-sensitivity check index; NS = non significant. Values expressed as mean ± SEM.

### Liver Triglyceride Content, Adiposity and Insulin Sensitivity

Insulin resistant animals had a ∼3 fold increase in TG content (p = 0.038) compared to control baboons (Figure 1-A). Fasting plasma insulin more strongly correlated with TG content in liver (R^2^ = 0.755, p<0.0001) than QUICKI which also showed a significant correlation with TG content (R^2^ = 0.628, p<0.0001; Figure 1 B–D). Increased leptin concentrations were present in the IR (Insulin Resistant) group as compared to the IS (Insulin Sensitive) group and a strong correlation was observed between plasma leptin levels and liver TG content in the whole study population (R^2^ = 0.599, p<0.0001; Figure 1-E and F). Peripheral (muscle) insulin sensitivity also significantly correlated with liver TG content (R = −0.461, p = 0.046). Liver TG content positively correlated also with plasma triglycerides (R = 0.455, p = 0.043), adipose tissue insulin resistance index (R = 0.799, p = <0.0001) and abdominal circumference (R = 0.619, p = 0.003) as indicated in Table 2. IGF-1 circulating levels were ∼15% lower in the IR group as compared to the IS, although this difference was not statistically significant (Figure S1). IGF-1 levels correlated negatively with circulating TG and TG/HDL ratio, but not with BMI, Waist, FPI, QUICKI, M/I, liver TG content, liver long chain saturated fatty acyl CoAs (LC-SFAs) and long chain polyunsaturated fatty acyl CoAs (LC-PUFAs) (Table S3).

**Table 2:** Correlations between liver TG content and long chain fatty acyl CoA with clinical and biochemical markers in baboons.

Liver TG Content	Coefficient of Correlation	*P*
Waist	0.619	0.0028
FPI	0.869	<0.0001
BMI	0.681	0.0006
NEFA	0.528	0.019
Plasma TG	0.455	0.043
TG/HDL	0.539	0.013
Leptin	0.774	<0.0001
QUICKI	−0.699	0.0005
M/I	−0.461	0.046
**Saturated LCFACoA**		
Waist	0.318	NS(0.17)
FPI	0.475	0.033
BMI	0.416	NS(0.068)
NEFA	0.387	NS(0.10)
Plasma TG	0.280	NS(0.17)
TG/HDL	0.467	0.037
Leptin	0.443	0.049
QUICKI	−0.589	0.007
M/I	−0.123	NS(0.62)
**Unsaturated LCFACoA**		
Waist	0.389	0.027
FPI	0.438	0.05
BMI	0.515	0.019
NEFA	0.466	0.043
Plasma TG	0.320	NS(0.17)
TG/HDL	0.417	NS(0.067)
Leptin	0.377	NS(0.10)
QUICKI	−0.570	0.009
M/I	−0.382	NS(0.11)

Obesity and insulin resistance is significantly correlated with higher liver TG content, and deleterious long chain fatty acyl CoA profile in baboons. NEFA = non-esterified fatty acids; FPI = fasting plasma insulin; M/I = glucose uptake/steady state plasma insulin, TG = triglycerides; NS = non significant.

### Hepatic and Peripheral Insulin Sensitivity and Long Chain Fatty Acyl CoA Concentration in the Liver

**Figure 2:**
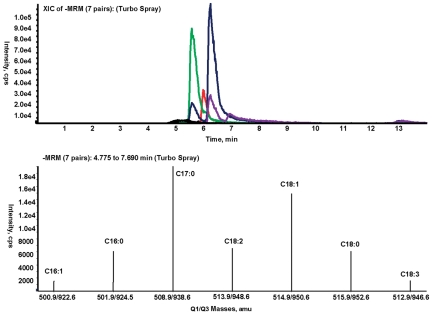
Quantification of hepatic long-chain fatty acyl CoA by ESI/MS/MS. Representative LC/MS/MS chromatogram (A) and negative ion pairs, [M-2H]^2−^/[M-H-80]^−^ (B) of long-chain fatty acyl CoA esters extracted from frozen baboon liver. Acyl-CoA esters were eluted on a C18 column with a gradient from 25% to 95% organic over 8 minutes of aqueous acetonitrile (2 mM ammonium acetate). LCCoA species were quantified from the integrated area under the curve of the corresponding ion pairs.

We measured the concentrations of each of the long-chain (C16 and C18, saturated and unsaturated) acyl CoA esters in the liver tissue of all 20 baboons using LC/MS/MS. A typical electrospray ionization (ESI)/MS/MS chromatogram and associated parent-daughter ion pairs of the saturated (C16∶0 and C18∶0) and unsaturated (C16∶1, C18∶1, C18∶2, and C18∶3) LC-FAs is shown in Figure 2. We found increased concentrations of long chain unsaturated fatty acyl CoA (LC-UFA) as compared to LC-SFA. We found no significant difference in the mean value of LC-SFA between groups (IS: 10.5±2.8, IR: 11.6±3.0 nmol/g-liver, p = 0.41), but a ∼40% increase in the concentration of LC-UFA in the liver of the IR baboons (IR: 22.4±7.2, IS: 15.9±5.4 nmol/g-liver, p = 0.034). This increase in the LC-UFA led to a higher (p = 0.002) ratio of LC-UFA to LC-SFA in the insulin resistant baboons compared to insulin sensitive controls (1.93±0.31 vs. 1.49±0.20 p = 0.002) as shown in Table 3.

**Table 3:** Tissue concentration of different saturated and unsaturated long chain fatty acyl CoA (LC-FACoA) in liver from obese insulin resistant (IR) baboons in comparison to insulin sensitive (IS) control group.

LC-FACoA (nmol/g tissue)	IR(n = 10)	IS(n = 10)	*P* value
16∶0 (Palmitic)	5±1.7	4.1±1.5	NS (0.22)
16∶ 1 (Palmitoleic)	1.5±0.4	1±0.4	0.044
18∶0 (Stearic)	6.5±1.3	6.3±1.3	NS (0.76)
18∶1 (Oleic)	10.8±4	7±2.8	0.029
18∶2 (Linoleic)	8.3±2.4	6.3±2	0.05
18∶3 (α-Linolenic)	1.8±0.6	1.5±0.6	NS (0.24)
MUFA	12.3±4.6	8±3	0.028
UFA	22.4±7	15.9±5	0.034
SFA	11.6±3	10.5±3	NS (0.41)
UFA/SFA	1.9±0.3	1.5±0.2	0.002
MUFA/SFA	1.04±0.2	0.76±0.2	0.002

MUFA = Monounsaturated fatty acyl CoA; UFA = Unsaturated fatty acyl CoA; SFA = Saturated fatty acyl CoA; NS = non significant. Values are expressed as mean ± SD.

On average, LC-UFA were ∼60% of total LCFA in the IS animals, while in IR animals the proportion was slightly but significantly increased to ∼66% (p<0.05) (Table 3). Both LC-UFAs and LC-SFAs significantly correlated with fasting insulin, QUICKI and TG/HDL ratio, but not with peripheral insulin sensitivity. LC-UFAs were also significantly associated with abdominal circumference, BMI and NEFA concentrations, and LC-SFAs were significantly associated with leptin concentrations (Table 2).

**Figure 3:**
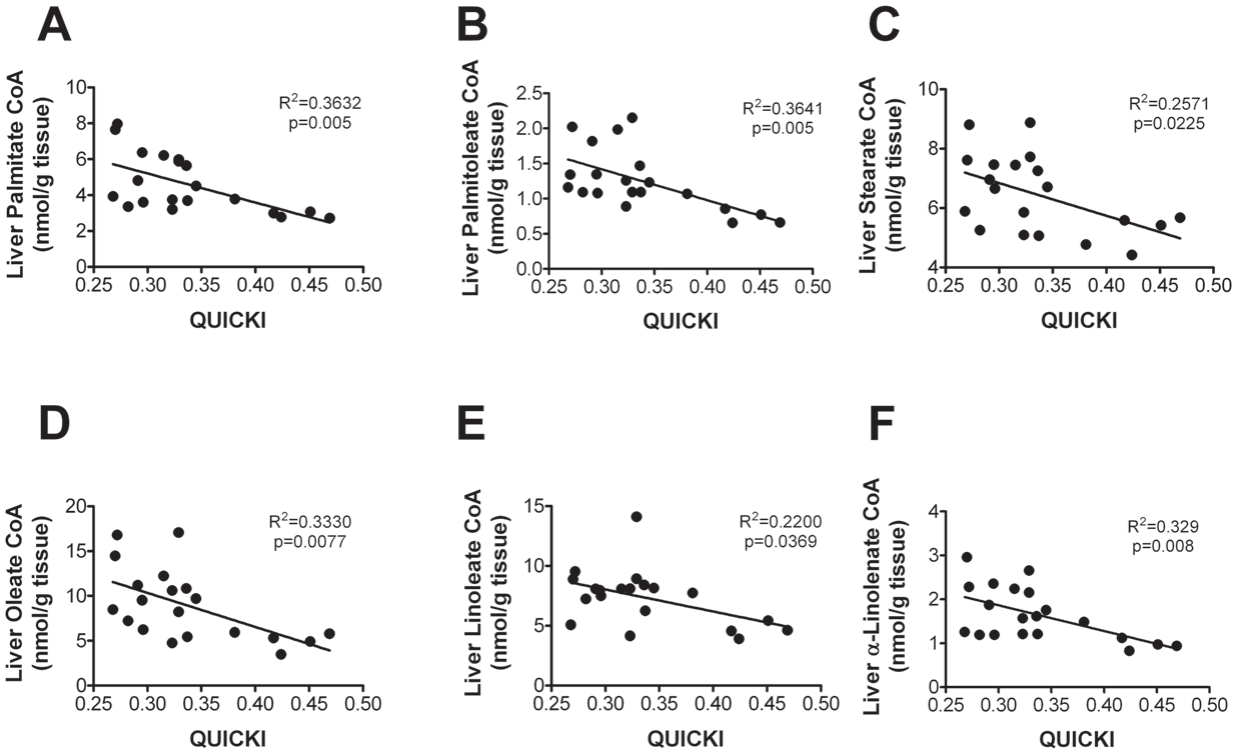
Increased LCFAs are associated with decreased insulin sensitivity in liver. Correlation between estimated hepatic insulin sensitivity and liver concentration of saturated (A and C), monounsaturated (B, D) and polyunsaturated (E and F) fatty acyl CoA in study population.

We also analyzed the contribution of specific LCFACoA to hepatic and peripheral insulin sensitivity. When we analyzed each fatty acid separately, we found that in all cases, the IR group had higher LCFACoA levels compared with the control IS group, although we found these differences to be statistically different for the monounsaturated fatty acids (Table 3). Interestingly, we found that in addition to these differences, all total saturated (palmitic and stearic), monounsaturated (palmitoleic and oleic) and polyunsaturated (linoleic and α-linolenic) acids negatively correlated with hepatic insulin sensitivity (Figure 3). Both saturated and polyunsaturated fatty acids correlated well also with markers of adiposity, particularly abdominal obesity, namely waist circumference, and to a lesser extent BMI. This suggests that intrahepatic accumulation of specific fatty acids as well as triglycerides, accounts for increased hepatic insulin resistance.

### Determinants of liver triglyceride content and hepatic insulin sensitivity

After adjusting for age, BMI, waist and triglyceride/HDL by using a step-wise approach, the linear regression model showed that the main determinant of hepatic insulin sensitivity (QUICKI) was the total liver TG content (partial R = −0.66, p = 0.005). Likewise, the main determinants of total TG content were QUICKI (partial R = −0.66, p = 0.005), age (partial R = 0.57, p = 0.02), and TG/HDL (partial R = 0.48, p = 0.06) explaining approximately 70% of the variation. (Adjusted R^2^ = 0.71, p = 0.0003). These results further emphasize the close relationship between central obesity and total fat content in baboon liver.

### Liver Histology and NAS Scoring

**Figure 4:**
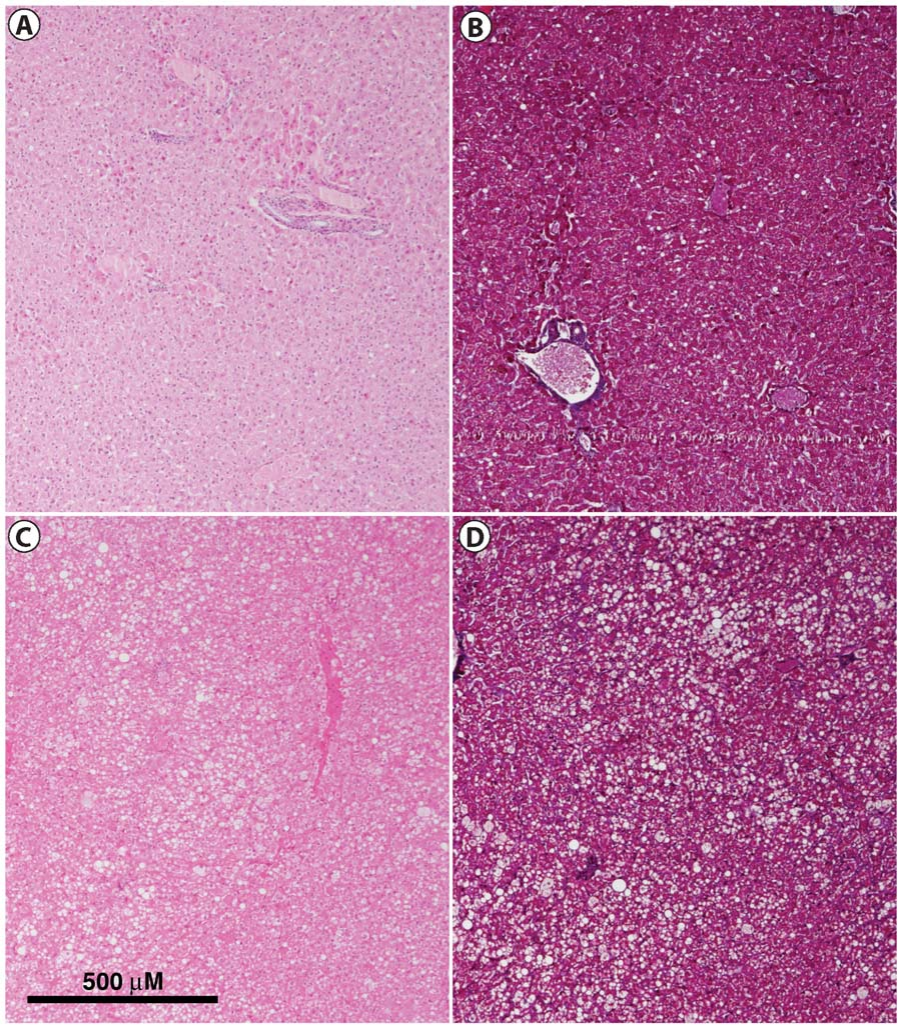
Steatosis in insulin resistant baboons. Histological sections of liver from a control insulin sensitive baboon (BMI = 16.5, QUICKI = 0.469, M = 16.25 mg•kg/min^−1^) with (A) Hematoxylin and Eosin (H&E) and (B) Masson's Trichrome stain showing normal parenchyma architecture; Histological sections from an insulin resistant baboon (BMI = 36.5, QUICKI = 0.268, M = 0.25 mg•kg/min^−1^) showing multifocal lipid accumulation and distension of hepatocytes characteristic of NAFLD with (C) H&E and (D) Masson's Trichrome stains respectively.

**Figure 5:**
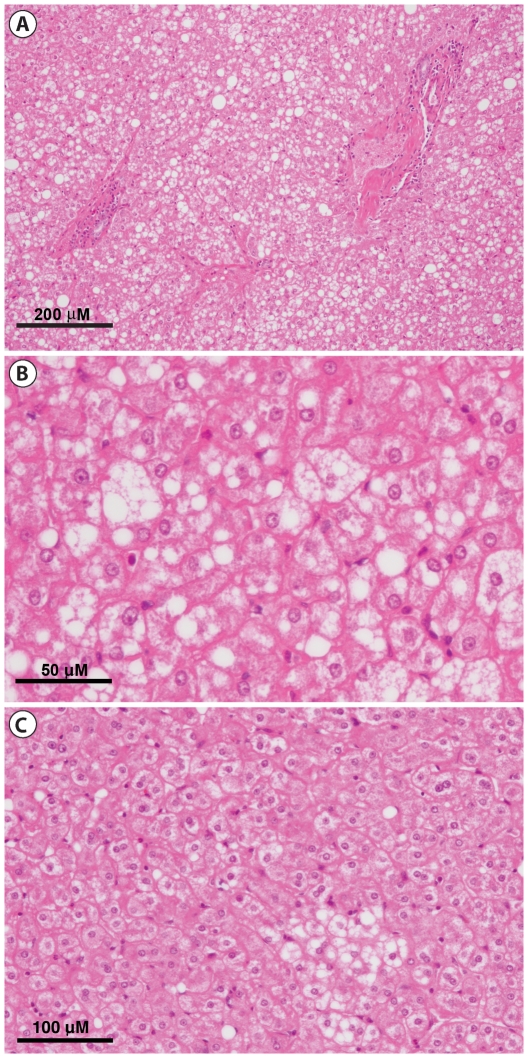
Macro steatosis, micro steatosis and ballooning in liver of insulin resistant baboons. Higher magnification of the liver from the insulin resistant baboon in Figure 4C and 4D showing discrete round lipid vacuoles within hepatocytes and ballooning (A&B). Focal area of minimal hepatic steatosis (C) in a different insulin resistant baboon ((BMI = 26.1, QUICKI = 0.291, M = 1.89 mg•kg/min^−1^).

Both the insulin sensitive and insulin resistant baboons had an overall normal liver architecture. Qualitative analysis of liver histology sections found a trend towards increased focal and disseminated hepatic steatosis in insulin resistant baboons when compared to insulin sensitive baboons, as determined by both QUICKI and clamp results (Figure 4 A–D). Overall, using the NAS scoring system we found characteristics compatible with NAFLD without meeting criteria for NASH (supported by the absence of liver fibrosis with Masson's trichrome staining) in obese insulin resistant baboons. Additionally, some insulin resistant animals showed changes in cell morphology including ballooning, a feature of progressing fatty liver disease (Figure 5 A–C). These morphological findings support the strong correlation between liver TG content and liver insulin sensitivity.

## Discussion

Hepatic triglyceride accumulation in NAFLD has been shown to be associated with an increased risk of diabetes and cardiovascular disease and is considered the hepatic manifestation of the metabolic syndrome X [28], [29], [30], [31]. In this study, taking advantage of the non-human primate baboon model of insulin resistance and obesity, we provide direct evidence for the increased accumulation of triglycerides in the liver and a detailed characterization of the specific fatty acids, possibly involved in the pathogenesis of NAFLD. We found that hepatic triglyceride accumulation, as well as increased concentrations of saturated and unsaturated fatty acyl CoAs, correlated well with hepatic insulin sensitivity.

Previous studies in murine models have provided insights into the metabolic profile of fatty liver disease [32]. The novelty of our study relies on the fact that in the same animal we analyzed hepatic histology, liver lipid composition, insulin sensitivity and their relationship with clinical and anthropometric measurements of adiposity. We believe that these findings represent an increase in knowledge since baboons are genetically very similar to humans, where the data available are insulin sensitivity and total hepatic and intramyocellular fat content by indirect measurements of magnetic resonance spectroscopy [33], [34], [35]. One limitation of our analysis is that we did not obtain direct measurements of hepatic insulin sensitivity with regard to glucose production since we did not utilize radio-labeled 3-H-glucose. However, given the relatively high insulin dose utilized in our clamp studies, we believe that most, if not all hepatic glucose production was suppressed [23]. Since direct biochemical evidence on hepatic TG content and fatty acyl CoA composition and their correlation with direct measures of insulin sensitivity are not available in humans, the study of non-human primate is highly relevant to understand the pathogenesis of the human disease counterpart. Also, some surrogate parameters have been established to analyze fatty acid metabolism in humans such as SCD-1 activity in VLDL sub-fractions [36], [37]. The underlying mechanisms by which obesity and insulin resistance cause NAFLD in humans are largely unknown. Several mechanisms described in murine models and cultured tissues could be of particular importance in the pathogenesis of hepatic steatosis and fibrosis. While leptin and insulin resistance are clearly associated with NAFLD, there is some indirect evidence supporting a primary role of increased delivery of non-esterified fatty acids to liver as a cause of hepatic steatosis in murine models. Interestingly, a number of cell culture studies have demonstrated that both insulin and leptin play an important role in stimulating fibrogenesis in the liver, and recently it has been demonstrated that fatty acids effectively trigger inflammatory cascades by interacting with toll-like receptors (TLR) [38], [39], [40], [41]. In fact, evidence suggests that specific length saturated, rather than unsaturated fatty acids (palmitic, 16∶0 and stearic, 18∶0) are related to this process providing a link between metabolism and inflammation [42].

Hepatic fat composition studies of biopsies obtained from morbid obese subjects undergoing bariatric surgery showed that the liver contained proportionally more palmitic (16∶0), stearic (18∶0), and long polyunsaturated fatty acids than adipose tissues. However, in that study only eight obese subjects were studied and no lean control subjects were reported [43]. Additional data from liver biopsies from patients affected by NAFLD undergoing bariatric surgery, showed parallel increases in gene expression of PPAR-γ and SREBP-1c which plays a key role in liver lipogenesis, in relation to markers of whole body insulin resistance [44]. Also in recent years, fatty acid binding proteins have been known to play a crucial role in obesity and glucose metabolism. The role of specific fatty acids has been previously evaluated in murine models, and to this extent several isomers of conjugated linoleic acid (18∶2) have been studied. While some isomers have shown a protective effect to prevent fatty liver, others have shown a clear deleterious effect [45], [46], [47], [48]. Due to the well known capacity of adipose tissue to produce hormones (adipocytokines) involved in the regulation of energy balance and insulin action we also measured the serum levels of leptin. Serum leptin was significantly lower in insulin sensitive as compared to insulin resistant baboons and showed a strong positive correlation with both FPG and liver TG content, and was inversely correlated to hepatic insulin clearance and QUICKI. Since leptin is directly associated with the fat mass content, we adjusted the correlation between liver TG content and QUICKI and FPI for fat content and body mass index, and these correlations remained statistically significant. This suggests that liver TG content is a determinant of hepatic insulin resistance, independent from body fat mass/degree of obesity. Interestingly, also an association between MCP-1, insulin resistance and circulating liver enzymes has been observed in baboons suggesting that they could be susceptible to development of NASH, although no histological data were provided in that study [49]. Our data in relation to the levels of long chain polyunsaturated fatty acids (18∶2 and 18∶3) contrasts with previous observations in other murine models where decreased concentrations of PUFA have been described in NAFLD. We consistently found higher levels of PUFA in obese insulin resistant baboons with hepatosteatosis. Stearoyl-CoA desaturase-1 (SCD-1) activity has been shown to play a central role in adaptation to hepatocellular injury in murine models of NAFLD and the ratio between monounsaturated to saturated NEFA has been hypothesized to reflect SCD-1 activity [50], [51]. However, SCD-1 activity and mRNA expression do not correlate with human liver fat content, although there was a very significant positive correlation between C18∶0/C18∶1 ratio and liver fat [52]. Our data are also in line with a previous report in patients with NASH and NAFLD in which an increase in polyunsaturated fatty acid liver concentration was found. However, in that report the relation between the liver TG content and fatty acid composition profile in relation with hepatic and peripheral insulin sensitivity as well as fat content and leptin levels were not evaluated [53]. While triglycerides are thought to represent an inert lipid species, LC-CoA esters are intermediary metabolites whose products directly affect energy production and synthesis of bioactive lipid species. LC-CoA esters lie at the crossroads of β-oxidation and mitochondrial energy production, and for the synthesis of lipid species (e.g. DAG and ceramides) that are proposed to induce hepatic insulin resistance, as demonstrated mainly, by studies in rodents [15], [54], [55], [56]. Although, the precise mechanisms behind the observed changes in LC-CoA concentrations reflect complex and competing processes, these changes can provide novel clues to altered mitochondrial energy production and lipid-mediated insulin resistance in humans.

Circulating IGF-1 levels are significantly lower in patients with NAFLD as compared to healthy controls [57]. Consistent with this recent observation, we also found a modest, although non significant decrease in circulating IGF-1 in IR as compared to IS animals, further emphasizing that non-human primates could be suitable models for NAFLD. In summary, hepatic triglyceride accumulation in obese baboons is a major determinant of hepatic insulin resistance. Furthermore increases in LC-SFAs as well LC-UFAs are also highly associated with markers of adiposity and decreased insulin sensitivity in liver and muscle. Overall, our results suggest that there are multiple abnormalities in fatty acid metabolism in obese IR baboons. Baboons are natural model for the study of insulin resistance and NAFLD, suited for mechanistic studies as well as to test novel dietary and pharmacological interventions. Future studies will address the molecular defects in hepatic insulin signaling and fatty acid accumulation in the liver, possibly in conjunction with lipidomic and proteomic analysis.

## Supporting Information

Figure S1 **Low levels of IGF-1 in insulin resistant baboons.** Circulating IGF-1 levels in IR (n = 10) and IS (n = 10) baboons.
(TIF)

Table S1 **Diet composition.**
(DOC)

Table S2 **Individual profiles (duplicate determinations) of various LC-FACoA concentrations in liver from obese insulin resistant vs. lean control (insulin sensitive) baboons.**
(DOC)

Table S3 **Correlations between circulating IGF-1 levels with clinical and biochemical indexes of insulin sensitivity, and liver TG, SFAs, PUFAs contents.**
(DOC)
