# Supplementary figures and images for: Coordinated Defects in Hepatic Long Chain Fatty Acid Metabolism and Triglyceride Accumulation Contribute to Insulin Resistance in Non-Human Primates
Source: PLoS One. 2011 Nov 18;6(11):e27617. doi: 10.1371/journal.pone.0027617 (PMC3220682; doi:10.1371/journal.pone.0027617)

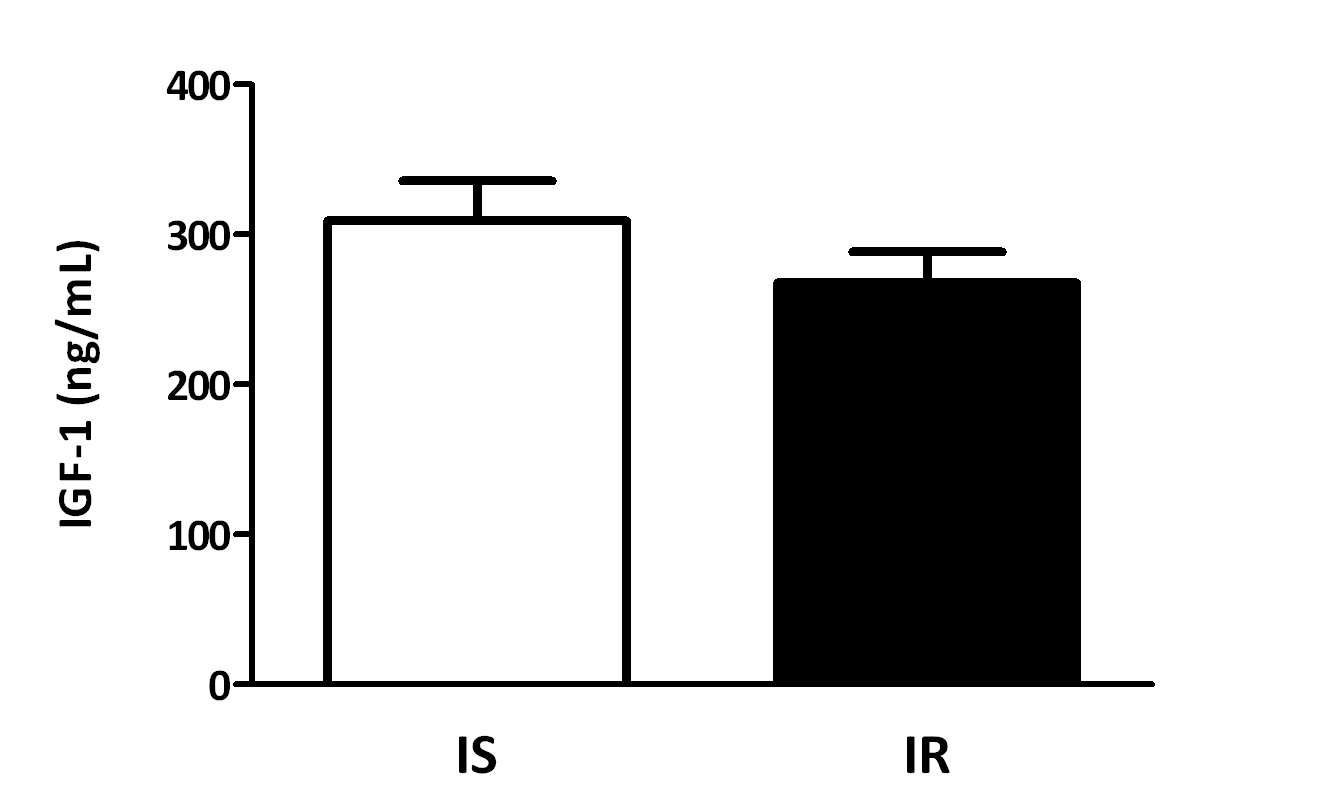

Supplement: Figure S1 — Low levels of IGF-1 in insulin resistant baboons. Circulating IGF-1 levels in IR (n = 10) and IS (n = 10) baboons. (TIF) [file pone.0027617.s001.tif]
