# Supplementary material for: Coordinated Defects in Hepatic Long Chain Fatty Acid Metabolism and Triglyceride Accumulation Contribute to Insulin Resistance in Non-Human Primates
Source: PLoS One. 2011 Nov 18;6(11):e27617. doi: 10.1371/journal.pone.0027617 (PMC3220682; doi:10.1371/journal.pone.0027617)
Supplement: Table S1 — Diet composition. (DOC) [file pone.0027617.s002.doc]

**Table S1.** Energy composition of diet.

|  | Kcal/g | %Kcal as fat | %Kcal as carbohydrate | %Kcal as sugar | %Kcal as protein |
| --- | --- | --- | --- | --- | --- |
| 5LE0 **^TM^** chow | 3.14 | 12.0 | 70.0 | 4.2 | 18.0 |
