# Supplementary material for: Coordinated Defects in Hepatic Long Chain Fatty Acid Metabolism and Triglyceride Accumulation Contribute to Insulin Resistance in Non-Human Primates
Source: PLoS One. 2011 Nov 18;6(11):e27617. doi: 10.1371/journal.pone.0027617 (PMC3220682; doi:10.1371/journal.pone.0027617)
Supplement: Table S2 — Individual profiles (duplicate determinations) of various LC-FACoA concentrations in liver from obese insulin resistant vs. lean control (insulin sensitive) baboons. (DOC) [file pone.0027617.s003.doc]

**Table S2**. Individual profiles (duplicate determinations) of various LC-FACoA concentrations in liver from obese insulin resistant vs. lean control (insulin sensitive) baboons.

|  | **Sample** |  |  |  |  |  |  |  |  |  |  |  | |  |  |  |
| --- | --- | --- | --- | --- | --- | --- | --- | --- | --- | --- | --- | --- | --- | --- | --- | --- |
|  | **Weight** | **nmole metabolite /g tissue** | | | | | | | | | | |  | |  |  |
| **ID** | **(mg)** |  | **C16:0** |  | **C16:1** |  | **C18:0** |  | **C18:1** |  | **C18:2** |  | | **C18:3** |  | **Total** |
| IR-1 | 37.0 |  | 3.74 |  | 1.26 |  | 5.88 |  | 10.83 |  | 8.07 |  | | 1.60 |  | 31.37 |
| IR-1 |  |  | 3.75 |  | 1.26 |  | 5.84 |  | 10.39 |  | 8.14 |  | | 1.54 |  | 30.92 |
|  |  |  |  |  |  |  |  |  |  |  |  |  | |  |  |  |
| IR-2 | 20.2 |  | 5.68 |  | 1.44 |  | 7.30 |  | 10.80 |  | 8.31 |  | | 1.55 |  | 35.09 |
| IR-2 |  |  | 5.61 |  | 1.49 |  | 7.21 |  | 10.87 |  | 8.51 |  | | 1.69 |  | 35.38 |
|  |  |  |  |  |  |  |  |  |  |  |  |  | |  |  |  |
| IR-3 | 23.6 |  | 3.42 |  | 1.06 |  | 5.42 |  | 7.12 |  | 7.31 |  | | 1.25 |  | 25.57 |
| IR-3 |  |  | 3.29 |  | 1.12 |  | 5.09 |  | 7.34 |  | 7.18 |  | | 1.13 |  | 25.15 |
|  |  |  |  |  |  |  |  |  |  |  |  |  | |  |  |  |
| IR-4 | 31.1 |  | 5.86 |  | 2.14 |  | 7.82 |  | 17.03 |  | 14.07 |  | | 2.62 |  | 49.53 |
| IR-4 |  |  | 5.92 |  | 2.16 |  | 7.63 |  | 17.13 |  | 14.14 |  | | 2.69 |  | 49.67 |
|  |  |  |  |  |  |  |  |  |  |  |  |  | |  |  |  |
| IR-5 | 46.5 |  | 7.72 |  | 1.34 |  | 7.74 |  | 14.52 |  | 8.90 |  | | 2.97 |  | 43.20 |
| IR-5 |  |  | 7.59 |  | 1.35 |  | 7.48 |  | 14.42 |  | 8.88 |  | | 2.94 |  | 42.67 |
|  |  |  |  |  |  |  |  |  |  |  |  |  | |  |  |  |
| IR-6 | 26.5 |  | 3.56 |  | 1.06 |  | 4.93 |  | 5.30 |  | 6.25 |  | | 1.19 |  | 22.30 |
| IR-6 |  |  | 3.82 |  | 1.13 |  | 5.20 |  | 5.58 |  | 6.26 |  | | 1.23 |  | 23.22 |
|  |  |  |  |  |  |  |  |  |  |  |  |  | |  |  |  |
| IR-7 | 23.0 |  | 3.57 |  | 1.09 |  | 4.71 |  | 5.96 |  | 7.77 |  | | 1.47 |  | 24.56 |
| IR-7 |  |  | 3.99 |  | 1.05 |  | 4.83 |  | 5.92 |  | 7.71 |  | | 1.50 |  | 25.01 |
|  |  |  |  |  |  |  |  |  |  |  |  |  | |  |  |  |
| IR-8 | 29.3 |  | 4.84 |  | 1.83 |  | 6.94 |  | 11.37 |  | 8.01 |  | | 1.86 |  | 34.86 |
| IR-8 |  |  | 4.78 |  | 1.81 |  | 6.97 |  | 11.01 |  | 8.15 |  | | 1.89 |  | 34.62 |
|  |  |  |  |  |  |  |  |  |  |  |  |  | |  |  |  |
| IR-9 | 45.9 |  | 8.02 |  | 2.04 |  | 8.66 |  | 16.81 |  | 9.40 |  | | 2.22 |  | 47.15 |
| IR-9 |  |  | 7.92 |  | 2.01 |  | 8.95 |  | 16.83 |  | 9.67 |  | | 2.35 |  | 47.73 |
|  |  |  |  |  |  |  |  |  |  |  |  |  | |  |  |  |
| IR-10 | 38.8 |  | 3.92 |  | 1.17 |  | 5.96 |  | 8.50 |  | 5.06 |  | | 1.28 |  | 25.90 |
| IR-10 |  |  | 3.94 |  | 1.15 |  | 5.81 |  | 8.49 |  | 5.10 |  | | 1.23 |  | 25.71 |

|  | **Sample** |  |  |  |  |  |  |  |  |  |  |  |  |  |  |
| --- | --- | --- | --- | --- | --- | --- | --- | --- | --- | --- | --- | --- | --- | --- | --- |
|  | **Weight** | **nmole metabolite /g tissue** | | | | | | | | | | |  |  |  |
| **ID** | **(mg)** |  | **C16:0** |  | **C16:1** |  | **C18:0** |  | **C18:1** |  | **C18:2** |  | **C18:3** |  | **Total** |
| IS-1 | 35.0 |  | 2.75 |  | 0.63 |  | 5.65 |  | 5.79 |  | 4.64 |  | 0.93 |  | 20.38 |
| IS-1 |  |  | 2.70 |  | 0.70 |  | 5.69 |  | 5.80 |  | 4.63 |  | 0.95 |  | 20.47 |
|  |  |  |  |  |  |  |  |  |  |  |  |  |  |  |  |
| IS-2 | 32.4 |  | 6.30 |  | 1.32 |  | 7.53 |  | 9.45 |  | 7.85 |  | 2.44 |  | 34.89 |
| IS-2 |  |  | 6.45 |  | 1.38 |  | 7.40 |  | 9.59 |  | 8.08 |  | 2.28 |  | 35.17 |
|  |  |  |  |  |  |  |  |  |  |  |  |  |  |  |  |
| IS-3 | 38.6 |  | 6.05 |  | 1.09 |  | 8.81 |  | 8.08 |  | 8.76 |  | 2.18 |  | 34.98 |
| IS-3 |  |  | 5.91 |  | 1.10 |  | 8.95 |  | 8.39 |  | 9.10 |  | 2.13 |  | 35.56 |
|  |  |  |  |  |  |  |  |  |  |  |  |  |  |  |  |
| IS-4 | 31.5 |  | 2.78 |  | 0.66 |  | 4.44 |  | 3.51 |  | 3.98 |  | 0.78 |  | 16.16 |
| IS-4 |  |  | 2.77 |  | 0.65 |  | 4.40 |  | 3.42 |  | 3.82 |  | 0.88 |  | 15.95 |
|  |  |  |  |  |  |  |  |  |  |  |  |  |  |  |  |
| IS-5 | 31.4 |  | 3.04 |  | 0.78 |  | 5.37 |  | 4.91 |  | 5.39 |  | 0.99 |  | 20.49 |
| IS-5 |  |  | 3.11 |  | 0.77 |  | 5.47 |  | 4.93 |  | 5.46 |  | 0.96 |  | 20.69 |
|  |  |  |  |  |  |  |  |  |  |  |  |  |  |  |  |
| IS-6 | 26.7 |  | 3.04 |  | 0.92 |  | 5.15 |  | 4.74 |  | 4.18 |  | 1.20 |  | 19.22 |
| IS-6 |  |  | 3.36 |  | 0.87 |  | 5.04 |  | 4.76 |  | 4.14 |  | 1.23 |  | 19.39 |
|  |  |  |  |  |  |  |  |  |  |  |  |  |  |  |  |
| IS-7 | 34.9 |  | 3.06 |  | 0.82 |  | 5.57 |  | 5.40 |  | 4.60 |  | 1.16 |  | 20.60 |
| IS-7 |  |  | 2.93 |  | 0.90 |  | 5.60 |  | 5.24 |  | 4.53 |  | 1.09 |  | 20.29 |
|  |  |  |  |  |  |  |  |  |  |  |  |  |  |  |  |
| IS-8 | 23.7 |  | 3.57 |  | 1.08 |  | 6.47 |  | 6.23 |  | 7.58 |  | 1.14 |  | 26.07 |
| IS-8 |  |  | 3.64 |  | 1.08 |  | 6.85 |  | 6.25 |  | 7.43 |  | 1.24 |  | 26.49 |
|  |  |  |  |  |  |  |  |  |  |  |  |  |  |  |  |
| IS-9 | 26.0 |  | 6.11 |  | 1.96 |  | 7.36 |  | 12.08 |  | 8.11 |  | 2.11 |  | 37.73 |
| IS-9 |  |  | 6.32 |  | 2.01 |  | 7.54 |  | 12.38 |  | 8.05 |  | 2.37 |  | 38.67 |
|  |  |  |  |  |  |  |  |  |  |  |  |  |  |  |  |
| IS-10 | 28.3 |  | 4.42 |  | 1.18 |  | 6.75 |  | 9.74 |  | 8.24 |  | 1.66 |  | 32.00 |
| IS-10 |  |  | 4.60 |  | 1.28 |  | 6.66 |  | 9.68 |  | 8.07 |  | 1.85 |  | 32.15 |
