# Supplementary material for: Coordinated Defects in Hepatic Long Chain Fatty Acid Metabolism and Triglyceride Accumulation Contribute to Insulin Resistance in Non-Human Primates
Source: PLoS One. 2011 Nov 18;6(11):e27617. doi: 10.1371/journal.pone.0027617 (PMC3220682; doi:10.1371/journal.pone.0027617)
Supplement: Table S3 — Correlations between circulating IGF-1 levels with clinical and biochemical indexes of insulin sensitivity, and liver TG, SFAs, PUFAs contents. (DOC) [file pone.0027617.s004.doc]

**Table S3.** Correlations between circulating IGF-1 levels with clinical and biochemical indexes of insulin sensitivity, and liver TG, SFAs, PUFAs contents.

| **IGF-1** | **Coefficient of correlation** | ***P*** |
| --- | --- | --- |
| Waist | -0.219 | NS (0.35) |
| FPI | -0.113 | NS (0.64) |
| BMI | 0.123 | NS (0.61) |
| NEFA | -0.1 | NS (0.68) |
| Plasma TG | -0.46 | 0.042 |
| TG/HDL | -0.561 | 0.01 |
| Leptin | -0.1 | NS (0.691) |
| QUICKI | 0.121 | NS (0.611) |
| M/I | 0.158 | NS (0.506) |
| Liver TG Content | -0.164 | NS (0.491) |
| LC-SFAs | 0.1 | NS (0.673) |
| LC-PUFAs | 0.077 | NS (0.748) |

IGF-1 is significantly negatively correlated with lower plasma TG and TG/HDL in baboons. NEFA=non-esterified fatty acids; FPI=fasting plasma insulin; M/I=glucose uptake/steady state plasma insulin; TG=triglycerides; LC-SFAs=Long chain saturated fatty acyl CoA; LC-PUFAs=Long chain poly-unsaturated fatty acyl CoA; NS=Non-significant.
